# Associations of Caregiving Knowledge and Skills With Caregiver Burden, Psychological Well-Being, and Coping Styles Among Primary Family Caregivers of People Living With Schizophrenia in China

**DOI:** 10.3389/fpsyt.2021.631420

**Published:** 2021-05-26

**Authors:** Zonglei Zhou, Yao Wang, Ping Feng, Tongxin Li, Jacob Kraemer Tebes, Rongsheng Luan, Yu Yu

**Affiliations:** ^1^Department of Epidemiology and Health Statistics, West China School of Public Health and West China Fourth Hospital, Sichuan University, Chengdu, China; ^2^Xiangya School of Nursing, Central South University, Changsha, China; ^3^Chongqing Municipal Center for Disease Control and Prevention, Chongqing, China; ^4^Department of Social Medicine and Health Management, Xiangya School of Public Health, Central South University, Changsha, China; ^5^Department of Psychiatry, Yale School of Medicine, New Haven, CT, United States

**Keywords:** schizophrenia, caregiving knowledge and skills, caregiver burden, psychological well-being, coping styles, primary family caregivers

## Abstract

**Background:** There is a lack of clarity regarding the correlation of caregiving knowledge and skills with caregiving experiences of people living with schizophrenia (PLSs). To address this gap, this comprehensive study examines the relationships of caregiving knowledge and skills to the primary family caregiver's experiences of burden, psychological well-being (stress, anxiety, depression, caregiving rewarding feelings), and coping styles in China.

**Methods:** A total of 395 primary family caregivers of PLSs were enrolled in a cross-sectional study between May 2019 and September 2019. Each family caregiver was independently assessed on caregiving knowledge and skills, caregiver burden, and psychological well-being, as well as coping styles.

**Results:** A higher level of caregiving knowledge and skills was positively correlated with less stress (*b* = −0.48, *P* < 0.001), anxiety (*b* = −0.23, *P* = 0.029), depression (*b* = −0.29, *P* = 0.013), and more caregiving rewarding feelings (*b* = 0.54, *P* < 0.001). Also, caregivers with more knowledge and skills were more inclined to adopt positive coping strategies (*b* = 0.44, *P* < 0.001). Despite these differences, caregivers with different levels of caregiving knowledge and skills reported comparable caregiver burden (*b* = 0.11, *P* = 0.705) and the use of a passive coping style (*b* = 0.10, *P* = 0.169).

**Conclusion:** Caregiving knowledge and skills are a reliable predictor of psychological well-being and active coping among the primary family caregivers of PLSs. These findings inform the development of psychoeducational interventions to support family caregivers of PLSs.

## Introduction

With a prevalence of 1–3% worldwide (1), schizophrenia is a psychiatric disorder characterized by profound disturbance in language, perception, thinking, and frequent psychotic experiences (2). People living with schizophrenia (PLSs) are characterized by positive symptoms such as delusions and hallucinations (3). They may also present negative symptoms reflected in alteration of motivation and volition, such as social withdrawal, difficulty in maintaining interpersonal relationships, apathy, and anhedonia (4–6). PLSs may also have other accompanying symptoms, such as cognition impairment and emotional disorder (e.g., mania and depression) (7, 8). Schizophrenia can occur at different stages of life course, while the risk varies across different age groups (9). Reports have shown that the prevalence of schizophrenia changes with age in an inverted U-shape, with the highest value at around 40 years of age. Globally, PLSs aged 25–54 years are estimated to account for 70.8% of total cases. There is no sexual difference in the prevalence (10). PLSs usually have reduced life expectancy in comparison with individuals from the general population (11). This disorder afflicts both PLSs and their family caregivers (12), particularly in countries with insufficient mental health resources and community health service systems (13). In China, more than 90% of PLSs live with their family members and receive family care because of insufficient community health resources (14).

Caring for a family member with schizophrenia requires considerable time, energy, and money from the family caregivers over a prolonged period, which may result in increased caregiver burden and psychological distress (15, 16). Numerous studies have demonstrated a high level of burden among caregivers of PLSs (12, 15, 17, 18) and psychological distress, such as stress, anxiety, and depression (19). For instance, more than half of caregivers report a moderate to severe level of caregiver burden (18), whereas the prevalence of psychological distress is as high as nearly 80% (1, 20). Notably, high levels of caregiver burden and psychological distress among caregivers may also impair the mental health of PLSs (21), who tend to experience physical or verbal domestic violence from their distressed caregivers (22, 23).

Despite significant caregiver burden and distress, there are also reports of positive impacts of caregiving (24, 25). Caring for PLSs may lead to positive transformations among caregivers (26, 27) and promote positive caregiving rewarding feelings, such as enhanced self-satisfaction, self-esteem, self-confidence, and self-affirmation (28, 29). Moreover, caring for PLSs can lead to improvements in stress-coping styles of caregivers (30), which are behavioral coping strategies adopted by individuals to resolve adversities and stressors (31). Evidence has shown that active coping is also associated with better health outcomes (32).

Over the past few decades, various interventions have been developed to support family caregivers of PLSs, of which psychoeducation is the most common and widely shown to be effective (33). Psychoeducation provides PLSs and family caregivers information about strategies and resources to promote the goals of treatment and rehabilitation (33). For caregivers, the key element of psychoeducation lies in improving knowledge and skills in caregiving (34). However, inconsistent findings about the correlations between caregiving knowledge and skills and caregiver burden have been reported by Sefasi et al. (35) and Jagannathan et al. (15). Sefasi et al. found caregivers of PLSs with more knowledge tended to bear greater burden, whereas Jagannathan et al. suggested there was no relationship of caregiving knowledge with burden. To date, no quantitative studies exist that examine the associations of caregiving knowledge and skills with psychological well-being (stress, anxiety, depression, caregiving rewarding feelings) and coping styles (active coping) among the primary family caregivers of PLSs in China.

The theoretical background of this study refers to the stress process model proposed by Pearlin et al. and their assumption that stress results from an interaction of two domains: stressors and mediators (36). Each of these domains can be divided into a series of subparts that have been intensively studied and developed in recent years. According to this model, it can be inferred that stress perceived by caregivers is partially mediated by coping and individual appraisal. Elevated caregiving knowledge and skills help enhance caregivers' ability to cope with adversity and enable them to adopt active coping strategies to address current dilemmas, thus contributing to the reduction of stress (37). Individual appraisal involves subjective assessment on controls of adversity and self-worth. A high level of caregiving knowledge and skills enables caregivers to resolve difficulties in the process of caring for PLSs, thus contributing to increased feelings of personal accomplishment and self-efficacy (38, 39). Furthermore, caregiving knowledge itself is a mediator in the path between stressors and burden (36, 40). Therefore, in the conceptual framework of this study, we assumed that caregiving knowledge and skills may be associated with caregiving experiences (caregiver burden, stress, coping styles, etc.) in a direct or indirect path.

The aim of this study was to evaluate the relationships between caregiving knowledge and skills and a range of caregiving experiences among primary family caregivers of PLSs in China.

## Methods

### Participants and Procedure

This cross-sectional study using one-stage cluster sampling was conducted in 12 community health centers of Changsha City, Hunan Province. The targeted population for this study was the primary family caregivers of PLSs who were registered in the “686 Program,” China's largest demonstration project in mental health services (41, 42). For each person living with schizophrenia, only one primary family caregiver was enrolled in this study based on closeness with PLSs and direct involvement in caregiving. The inclusion criteria were as follows: (1) aged over 18 years; (2) caring for individuals diagnosed with schizophrenia according to the criteria of Chinese Classification of Mental Disorders Version 3 (43) or the *International Classification of Diseases, Tenth Revision* (44); (3) living with PLSs for over 2 years and taking major responsibility for caregiving; (4) able to understand, read, and communicate with investigators in Chinese. The exclusion criteria included (1) caring for PLSs who received a diagnosis other than schizophrenia; (2) unable to speak Chinese; and (3) lack of literacy, or having serious physical or mental diseases that made them unable to communicate effectively with others.

Data were collected by well-trained researchers from May 2019 to September 2019. First, all participants were informed about the purpose of the study and provided written informed consent before participating. Second, the overall content of questionnaires was introduced to each eligible participant, and they were invited to complete the questionnaires (see assessment below) by face-to-face interviews. Next, a quality-control investigator checked the answers to ensure accuracy, integrity, and consistency. A total of 414 primary family caregivers volunteered to participate in this study, of which 395 participants completed the questionnaire (response rate = 95.41%). No significant difference was detected between individuals who completed and those who did not complete the survey in terms of gender, age, marriage, education, and employment.

The institutional review board of the Xiangya School of Public Health of Central South University approved the protocol of this study. Participants who completed the questionnaire were reimbursed with RMB 20 yuan in cash ($3).

### Data Collection

#### Sociodemographic Data

Sociodemographic data of the primary family caregivers were collected by face-to-face interviews, including sex, age, marital status, education, employment status, financial circumstances (annual income per household), length of caregiving, and the relationship between PLSs and the primary caregivers. Besides, whether there were any co-caregivers, additional dependents, and physical illnesses were also recorded.

#### Caregiving Knowledge and Skills

The understanding of schizophrenia and mastery of caregiving skills were evaluated using the Knowledge and Skills of Caregiving Scale (KSCS). The KSCS is a five-item questionnaire, which was originally designed according to the survey on the demands of mental health knowledge among the Chinese caregivers of PLSs (45–47). Items are scored on a 4-point Likert scale from 0 (do not understand) to 3 (totally understand) to measure the caregiver's understanding of the following aspects: the symptoms of schizophrenia, medication and its side effects, the care of PLSs, and management of symptoms. The total KSCS score ranges from 0 to 15, with a higher score demonstrating higher knowledge of schizophrenia and better skills in caregiving. The detailed contents of the KSCS are illustrated in Supplementary Table 1. The KSCS showed good internal consistency in the current study, with a Cronbach α of 0.87.

#### Caregiver Burden

Caregiver burden was evaluated using the Zarit Burden Interview (ZBI) (48). The ZBI consists of 22 items, and each item is rated on a 5-point response scale from 0 (never) to 4 (nearly always), except for the final item evaluating global burden from 0 (not at all) to 4 (extremely). The total score ranges from 0 to 88, and higher scores are indicative of higher burden perceived by caregivers. This instrument was first translated into Chinese by Lu et al. (49) in 2009 and found to be reliable and valid in evaluating caregiver burden (50, 51). The ZBI also demonstrated excellent internal consistency in this sample, with a Cronbach α of 0.92.

#### Stress

Perceived stress was assessed using the 10-item Perceived Stress Scale (PSS-10) (52). This instrument covers two domains: perceived helplessness (six items) and perceived self-efficacy (four items) (53, 54). Each item is scored on a 5-point Likert scale from 0 (never) to 4 (often); the total score ranges from 0 to 40, with higher scores indicating greater perceived stress. First translated into Chinese by Yang et al. (55) in 2003, the PSS-10 is reliable in evaluating perceived stress (56, 57). In this study, the PSS-10 exhibited acceptable internal consistency, with a Cronbach α of 0.79.

#### Anxiety

The seven-item Generalized Anxiety Disorder Scale (GAD-7) (58) was used to reflect the anxiety symptoms of primary family caregivers in the past 2 weeks. Items rated on a 4-point Likert scale from 0 (not at all) to 3 (nearly every day) are summed into a total score, which ranges from 0 to 21, and a cutoff score of 10 has been suggested to differentiate anxiety and non-anxiety (59). The GAD-7 was first translated into Chinese by He et al. (60) in 2010 and is a valid and reliable measure of anxiety among the Chinese population (61). In this study, the GAD-7 demonstrated good internal consistency with a Cronbach α of 0.95.

#### Depression

The depression symptoms of primary family caregivers were screened using the self-reported Patient Health Questionnaire (PHQ-9) (62). The PHQ-9 consists of nine items rated on a 4-point Likert scale from 0 (not at all) to 3 (nearly every day) to evaluate the frequency of depression symptoms in the preceding 2 weeks. The total score ranges from 0 to 27, with higher scores indicative of more depression symptoms. The PHQ-9 was first translated into Chinese by Yeung et al. (63) in 2008 and showed good validity and reliability in Chinese context (64). In the present study, the Cronbach α for the PHQ-9 was 0.93.

#### Caregiving Rewarding Feelings

The Caregiving Rewarding Feelings (CRF) scale was applied to evaluate positive feelings about caregiving (65). Thirty individuals with schizophrenia and their primary caregivers were qualitatively interviewed for the initial development of the CRF, pretesting, and Delphi method for further revision and then a test with a larger sample to confirm its validity and reliability. The detailed process of development and validation of the CRF has been illustrated elsewhere (65). The CRF is a 12-item questionnaire developed in a Chinese context, and each item is scored on a 4-point Likert scale (0 = “never” to 3 = “nearly always”) to assess whether caring for PLSs makes the primary caregivers “become more loving and patient,” “feels more worthy,” “be more active and optimistic,” “have stronger sense of responsibility,” etc. The total score ranges from 0 to 36, with a higher score suggesting better positive feelings. In this study, the CRF demonstrated excellent internal consistency with a Cronbach α of 0.96.

#### Coping Styles

The Simplified Coping Style Questionnaire (SCSQ) was used to measure a caregiver's coping style (66). This measure is a 4-point Likert questionnaire (0 = “never” to 3 = “nearly always”) that comprises 20 items classified into two dimensions: active coping (12 items) and passive coping (eight items). Active coping mainly reflects one's active coping preference when encountering difficulties, such as “working out several different ways to solve the problem.” Passive coping mainly reflects one's passive coping tendency when encountering difficulties, such as “relying on others to solve the problem.” The total score ranges from 0 to 60, and a higher score for each dimension indicates a higher possibility that the participant would adopt the corresponding coping style. Good reliability and validity of the SCSQ in a Chinese context were reported by Xie (66), and a Cronbach α of 0.88 for the total scale, 0.90 for the subscale of active coping, 0.72 for the subscale of passive coping were detected in the current study.

### Sample Size

Sample size was calculated using the correlation power and sample size calculation in G^*^Power. Assuming a correlation of 0.2 between caregiving knowledge and skill and stress, a *Z* value of 1.98 at a confidence interval of 95%, and allowable α error of 5%, we needed a sample size of 319. Considering a rejection or loss-to-follow-up rate of 10%, we expanded our final sample size to 350. Our study recruited 395 participants, which satisfies the sample size requirement with a power of 95%.

### Statistical Analyses

All statistical analyses were performed using STATA software version 16.0. The categorical variables were presented as counts (percentages), whereas the continuous variables were expressed as mean (SD) or median [interquartile range (IQR)]. Normality of data was determined using frequency distributions (histogram), Kolmogorov–Smirnov test, and skewness and kurtosis statistics. The difference of KSCS scores in different sociodemographic groups was checked using Mann–Whitney *U*-test or Kruskal–Wallis *H* test. The correlations between knowledge and skills of caregiving and various types of caregiving experiences (caregiver burden, stress, anxiety, depression, caregiving rewarding feelings, coping styles) were examined by Spearman rank correlation analysis. Adjusted by potential confounders (gender, age, marital status, education, employment, annual income, etc.), several multivariate linear regressions were separately performed to examine the associations between KSCS score and seven types of caregiving experiences among primary family caregivers. Potential multicollinearity between predictor variables was checked by variance inflation factor (VIF), with VIF > 5 indicating collinearity (13). The difference was considered statistically significant at *P* < 0.05.

## Results

### General Characteristics and Group Comparison of KSCS Score

The sociodemographic characteristics of the participants are described in Table 1. There were slightly more females (55%) than males (45%). Most of the caregivers were older than 60 years (64%), were married (76%), had a middle school education (60%), and were unemployed (87%), with an annual family income of <10,000 yuan (60%). The largest proportion of caregivers were parents (58%). Most caregivers had been caring for PLSs for over 10 years (81%) and without co-caregivers (56%). Sixty percent of caregivers had no additional dependents and had physical illness such as hypertension, diabetes mellitus, and cardiovascular diseases.

**Table 1:** Sociodemographic characteristics of the caregivers and comparison of KSCS scores between groups.

**Variables**		**No. (%)**	**KSCS score**		
			**Median (IQR)**	***Z*/*H***	***P***
Gender	Male	178 (45.06)	10 (8–11)	−0.572	0.567
	Female	217 (54.94)	10 (8–12)		
Age (years)	18–44	38 (9.62)	10 (7–11)	5.151	0.076
	45–59	105 (26.58)	10 (9–12)		
	≥60	252 (63.80)	10 (8–11)		
Marital status	Not married^†^	93 (23.54)	10 (7–11)	−1.981	**0.048**
	Currently married^††^	302 (76.46)	10 (8–12)		
Education	Primary	123 (31.14)	10 (7–11)	6.141	**0.046**
	Middle	238 (60.25)	10 (9–12)		
	High	34 (8.61)	10 (8.75–10)		
Employment status	Unemployed	342 (86.58)	10 (8–11)	−0.807	0.420
	Employed	53 (13.42)	10 (9–13)		
Annual income (yuan/person)	<10,000	236 (59.75)	10 (8–11)	2.750	0.253
	10,000–19,999	112 (28.35)	10 (8–12)		
	≥20,000	47 (11.90)	10 (9–11)		
Length of caring (years)	<10	75 (18.99)	10 (9–11)	−0.567	0.570
	≥10	320 (81.01)	10 (8–11)		
Relationship to the PLSs	Parents	231 (58.48)	10 (8–11)	4.378	0.357
	Spouse	98 (24.81)	10 (8–13)		
	Children	23 (5.82)	10 (9–11)		
	Siblings	34 (8.61)	10 (7–10)		
	Others	9 (2.28)	10 (6–10)		
Co-caregiver	No	221 (55.95)	10 (8–12)	−0.991	0.322
	Yes	174 (44.05)	10 (8–11)		
Additional dependents	No	236 (59.75)	10 (8–11)	−0.109	0.913
	Yes	159 (40.25)	10 (8–12)		
Physical illness	No	156 (39.49)	10 (8–12)	−1.018	0.308
	Yes	239 (60.51)	10 (8–11)		

*KSCS, Knowledge and Skill of Caregiving Scale; IQR, interquartile range; PLSs, people living with schizophrenia*.

† *Includes single, widowed, divorced, and separated*.

†† *Includes married and cohabited*.

*Values in bold font are statistically significant (P < 0.05)*.

The median KSCS score was 10, with an IQR of 8–11. We further compared the KSCS score between groups with different sociodemographic characteristics and found no significant difference except for marital status and education. Caregivers who were married, with higher educational attainment, had better caregiving knowledge and skills (*P* = 0.048, *P* = 0.046).

### Correlations of Knowledge and Skills With Caregiving Experiences

Table 2 summarizes the caregiving experiences concerning caregiver burden, psychological well-being, and coping styles, divided into negative and positive caregiving experiences. Negative caregiving experiences comprised caregiver burden (mean = 43.21), stress (mean = 18.72), anxiety (mean = 7.68), depression (mean = 8.30), and passive coping (mean = 11.80). Positive caregiving experiences consisted of caregiving rewarding feelings (mean = 26.20) and active coping (mean = 20.02).

**Table 2:** Caregiving impacts and Spearman correlation coefficients between outcomes and KSCS scores.

**Variables**	**Mean (SD)**	**Spearman correlation**	
		***r***	***P***
Subjective burden (ZBI)	43.21 (18.26)	0.008	0.878
Stress (PSS-10)	18.72 (6.84)	−0.210	**<0.001**
Anxiety (GAD-7)	7.68 (6.45)	−0.104	**0.039**
Depression (PHQ-9)	8.30 (7.26)	−0.167	**<0.001**
CRF	26.20 (9.11)	0.208	**<0.001**
Active coping (SCSQ subscale)	20.02 (7.72)	0.168	**<0.001**
Passive coping (SCSQ subscale)	11.80 (4.54)	0.004	0.930

*KSCS, Knowledge and Skills of Caregiving Scale; ZBI, Zarit Burden Interview; PSS-10, 10-item Perceived Stress Scale; GAD-7, seven-item Generalized Anxiety Disorder Scale; PHQ-9, nine-item Patient Health Questionnaire; CRF, Caregiving Rewarding Feelings; SCSQ, Simplified Coping Style Questionnaire; SD, standard deviation*.

*Values in bold font are statistically significant (P < 0.05)*.

Caregivers' knowledge and skills about caregiving were negatively correlated with stress (*r* = −0.210, *P* < 0.001), anxiety (*r* = −0.104, *P* = 0.039), and depression (*r* = −0.167, *P* < 0.001), and positively associated with caregiving rewarding feelings (*r* = 0.208, *P* < 0.001) and active coping (*r* = 0.168, *P* < 0.001). But caregiving knowledge and skills did not correlate with either caregiver burden or passive coping, despite a small trend in favor of a positive correlation.

### Multivariate Linear Regression

Table 3 shows the results of several multivariate linear regressions of caregiving knowledge and skills on seven types of caregiving experiences (caregiver burden, stress, anxiety, depression, caregiving rewarding feelings, active coping, passive coping). After controlling for covariates, caregiving knowledge and skills were still significantly negatively associated with stress (*b* = −0.48, *P* < 0.001), anxiety (*b* = −0.23, *P* = 0.029), and depression (*b* = −0.29, *P* = 0.013), as well as positively associated with caregiving rewarding feelings (*b* = 0.54, *P* < 0.001). Besides, primary caregivers with more caregiving knowledge and skills were more inclined to adopt positive coping strategies (*b* = 0.44, *P* < 0.001). No significant relationship was detected between caregiving knowledge and skills with either caregiver burden (*b* = 0.11, *P* = 0.705) or passive coping (*b* = 0.10, *P* = 0.169).

**Table 3:** Multivariate linear regression analysis of KSCS scores predicting caregiving impacts.

**Variables**		**Caregiver burden (ZBI)**	**Stress (PSS-10)**	**Anxiety (GAD-7)**	**Depression (PHQ-9)**	**Caregiving rewarding feelings (CRF)**	**Active coping style (SCSQ subscale)**	**Passive coping style (SCSQ subscale)**
KSCS score		0.11 (−0.45, 0.66)	−0.48^***^ **(−0.70**, **−0.27)**	−0.23^*^ **(−0.43**, **−0.02)**	−0.29^*^ **(−0.52**, **−0.06)**	0.54^***^ **(0.25, 0.84)**	0.44^***^ **(0.20, 0.68)**	0.10 (−0.04, 0.25)
Gender	Male	Ref	Ref	Ref	Ref	Ref	Ref	Ref
	Female	1.67 (−1.99, 5.33)	0.55 (−0.84, 1.95)	0.25 (−1.08, 1.57)	0.76 (−0.75, 2.27)	−0.89 (−2.80, 1.03)	0.63 (−0.94, 2.20)	−0.13 (−1.10, 0.85)
Age		−0.03 (−0.23, 0.18)	0.01 (−0.07, 0.09)	0.00 (−0.08, 0.07)	−0.01 (−0.09, 0.08)	−0.09 (−0.20, 0.02)	−0.09^*^ **(−0.18**, **−0.00)**	−0.02 (−0.07, 0.03)
Marital status	Not married	Ref	Ref	Ref	Ref	Ref	Ref	Ref
	Currently married	1.11 (−3.35, 5.56)	0.58 (−1.11, 2.28)	1.01 (−0.60, 2.63)	−0.52 (−2.36, 1.32)	0.77 (−1.55, 3.10)	−0.10 (−2.01, 1.81)	0.10 (−1.08, 1.29)
Education	Primary	Ref	Ref	Ref	Ref	Ref	Ref	Ref
	Middle	5.98^**^ **(2.12, 9.85)**	0.34 (−1.13, 1.81)	0.75 (−0.65, 2.15)	0.72 (−0.87, 2.32)	2.58^*^ **(0.55, 4.61)**	2.78^**^ **(1.12, 4.44)**	0.56 (−0.47, 1.59)
	High	4.46 (−2.65, 11.58)	−1.04 (−3.74, 1.67)	0.98 (−1.60, 3.55)	0.99 (−1.94, 3.93)	5.53^**^ **(1.81, 9.25)**	5.33^**^ **(2.28, 8.38)**	0.34 (−1.55, 2.24)
Employment	Unemployed	Ref	Ref	Ref	Ref	Ref	Ref	Ref
status	Employed	−3.95 (−9.60, 1.71)	−1.26 (−3.41, 0.89)	−1.28 (−3.33, 0.76)	−1.80 (−4.13, 0.53)	−0.04 (−2.99, 2.91)	−0.55 (−2.97, 1.87)	−0.56 (−2.07, 0.94)
Annual income (yuan/person)		−0.00^*^ **(−0.00**, **−0.00)**	−0.00 (−0.00, 0.00)	−0.00^**^ **(−0.00**, **−0.00)**	−0.00^**^ **(−0.00**, **−0.00)**	0.00 (−0.00, 0.00)	0.00 (−0.00, 0.00)	−0.00^*^ **(−0.00**, **−0.00)**
Length of caring (years)		−0.01 (−0.17, 0.14)	0.03 (−0.03, 0.09)	0.01 (−0.05, 0.06)	0.00 (−0.07, 0.06)	−0.07 (−0.15, 0.01)	−0.06 (−0.13, 0.01)	0.00 (−0.05, 0.04)
Relationship to the	Parents	Ref	Ref	Ref	Ref	Ref	Ref	Ref
PLSs	Spouse	–9.02^***^ **(**–**13.94**, –**4.09)**	−1.81 (−3.68, 0.07)	–2.41^**^ **(**–**4.20**, **−0.63)**	−1.15 (−3.19, 0.88)	−0.96 (−3.53, 1.61)	−0.20 (−2.31, 1.91)	−1.14 (−2.45, 0.17)
	Children	−12.04^**^ **(−20.90**, **−3.19)**	−0.65 (−4.02, 2.72)	−3.00 (−6.20, 0.20)	−3.47 (−7.12, 0.18)	2.27 (−2.36, 6.89)	0.80 (−3.00, 4.60)	−2.00 (−4.35, 0.36)
	Siblings	−6.57^*^ **(−13.08**, **−0.05)**	−1.59 (−4.07, 0.89)	−1.20 (−3.55, 1.16)	−0.82 (−3.50, 1.87)	0.15 (−3.30, 3.60)	0.72 (−2.07, 3.52)	−0.21 (−1.94, 1.52)
	Others	−18.42^**^ **(−30.04**, **−6.81)**	−0.45 (−4.87, 3.97)	−2.03 (−6.24, 2.17)	1.82 (−2.97, 6.61)	−0.85 (−6.91, 5.22)	−5.10^*^ **(−10.09**, **−0.12)**	−0.94 (−4.03, 2.15)
Co–caregiver	No	Ref	Ref	Ref	Ref	Ref	Ref	Ref
	Yes	−3.45 (−7.16, 0.26)	−2.09^**^ **(−3.50**, **−0.68)**	−2.36^**^ **(−3.70**, **−1.02)**	−1.15 (−2.68, 0.38)	2.26^*^ **(0.32, 4.20)**	−0.30 (−1.29, 1.89)	−1.04^*^ **(−2.03**, **−0.06)**
Additional	No	Ref	Ref	Ref	Ref	Ref	Ref	Ref
dependents	Yes	6.66^***^ **(3.07, 10.25)**	1.53^*^ **(0.16, 2.89)**	1.70^*^ **(0.40, 3.00)**	1.98^**^ **(0.50, 3.46)**	−1.32 (−3.20, 0.56)	−0.97 (−2.51, 0.57)	0.23 (−0.73, 1.18)
Physical illness	No	Ref	Ref	Ref	Ref	Ref	Ref	Ref
	Yes	4.33^*^ **(0.43, 8.22)**	1.15 (−0.33, 2.63)	0.83 (−0.58, 2.24)	0.67 (−0.93, 2.28)	−0.96 (−3.00, 1.08)	0.52 (−1.15, 2.19)	0.37 (−0.66, 1.41)
*R*^2^		16.41%	13.84%	12.28%	10.04%	15.81%	14.07%	4.65%
VIF	Mean (range)	1.30 (1.05–2.12)	1.30 (1.05–2.12)	1.30 (1.05–2.12)	1.30 (1.05–2.12)	1.31 (1.06–2.12)	1.30 (1.05–2.12)	1.30 (1.05–2.12)

*KSCS, Knowledge and Skills of Caregiving Scale; ZBI, Zarit Burden Interview; PSS-10, 10-item Perceived Stress Scale; GAD-7, seven-item Generalized Anxiety Disorder Scale; PHQ-9, nine-item Patient Health Questionnaire; CRF, Caregiving Rewarding Feelings; SCSQ, Simplified Coping Style Questionnaire*.

*VIF, variance inflation factor. Collinearity existed if VIF >5, and there was little possibility of multicollinearity as all VIF <5*.

*Values in bold font are statistically significant (P < 0.05)*.

* *P < 0.05*,

** *P < 0.01*,

*** *P < 0.001*.

## Discussion

To our knowledge, this is the first study that investigates comprehensively the associations of caregiving knowledge and skills with caregiver burden, psychological well-being, and coping styles among primary family caregivers of PLSs in China. Although no relationship was found between caregiving knowledge and skills and caregiver burden, caregiving knowledge and skills were negatively associated with stress, anxiety, and depression and positively associated with caregiving rewarding feelings. In addition, caregivers with more caregiving knowledge and skills were more inclined to adopt positive coping strategies, but not passive coping strategies. These findings add further evidence about the positive associations of caregiving knowledge and skills with a range of health outcomes and inform future psychoeducational interventions for improving caregiver outcomes.

Our study informs the relationship between caregiving knowledge and skills and caregiver burden. Some previous reports indicated that as the level of knowledge about schizophrenia increased, the perceived burden decreased (67, 68), whereas other studies showed the opposite (35). Consistent with previous research reported by Lim et al. (69) and Jagannathan et al. (15), our study showed that primary caregivers' knowledge about schizophrenia did not have any relationship to caregiver burden. Previous research suggested that avoidant coping may lead to increased caregiver burden (70), and the lack of association of caregiving knowledge and skills with caregiver burden in the current study may be due to the null association of caregiving knowledge and skills with passive coping. It is likely that coping style may mediate the relationship between caregiving knowledge and skills and caregiver burden, which warrants further study (71).

The current study also suggests that increased knowledge and skills of caregiving may relieve psychological distress, such as stress, anxiety, and depression. The stress process model argues that stress source, such as family conflicts and financial issues, is an important domain contributing to caregiver burden (36). Wan et al. (1) reported that lack of understanding of psychotic symptoms can lead to conflicts between caregivers and PLSs, which subsequently resulted in a high level of psychological distress among caregivers. Furthermore, caregivers with little knowledge may be more likely to hold false beliefs about schizophrenia, such as thinking that it is the result of bewitchment or drug abuse (72). When bewitchment is viewed as the cause of schizophrenia, caregivers usually hold pessimistic views (73) and lose hope for the recovery of PLSs, leading to psychological distress (35). Thus, increased knowledge and skills of caregiving may help caregivers change their attribution about the cause of schizophrenia so as to better understand the challenges faced by PLSs and take more active steps when crises occur (74). Improved caregiving knowledge and skills may also reduce caregivers' stigma associated with schizophrenia, which is known to be associated with depression and suicidal thoughts (75–77).

Under the framework of the stress process model, individual evaluation on self-worth is considered as a significant moderator of stress and is susceptible to subjective psychological factors. In this study, we demonstrated that improved caregiving knowledge and skills were associated with more caregiving rewarding feelings among the caregivers, an observation that may be explained, in part, by the Chinese culture. Under the influence of Confucianism, the dominant concept of collectivism is deeply rooted in the Chinese culture (13). Family members usually consider care for PLSs as their responsibility, and they may make every effort to facilitate the patient's recovery. A higher level of caregiving knowledge and skills allows them to assume more responsibility and provide better support for their loved one with schizophrenia, which may bring in a higher sense of self-achievement (38). A recent study with primary family caregivers of PLSs showed that improved knowledge and understanding of schizophrenia were effective in increasing their social interactions and promoting greater optimism for the future (74). Also, more knowledge about schizophrenia helps improve the caregiver's ability to monitor symptoms and initiate better collaboration with mental healthcare professionals, thus leading to improved self-efficacy (39).

In the stress process model, the interaction between stressors and psychological outcomes can be mediated by coping strategy (36). Similarly, a heuristic multivariate model proposed by Vitaliano et al. considers that burden results from three domains: stressor, vulnerability, and resource (78). In this model, passive coping is a risk factor for vulnerability. The current results showed that family caregivers with a higher level of caregiving knowledge and skills were more likely to adopt positive coping behaviors, which was related to better mental health (79). This finding also partially explained the phenomenon that a higher level of caregiving knowledge and skills predicted improved psychological well-being. For caregivers, increased knowledge and skills of caregiving may enhance individual confidence and competence in challenging difficulties (80), which is revealed in adopting active coping strategies. As noted above, caregiving knowledge and skills also correlated with hopefulness, which was found to be a predictor of problem-oriented coping (81). Besides, the stress–appraisal–coping paradigm is another theoretical framework for understanding the relationship between caregiving knowledge and stress (82). A better understanding of schizophrenia and caregiving may enable caregivers to reappraise the demands of caregiving, develop more effective strategies to cope with the problems related to caregiving, and better handle maladaptive behaviors of the PLSs, thus leading to reduced stress (74, 83).

### Limitations

The present study has several limitations. First, there was a potential selection bias as the participants in the current study were from the “686 Program,” who may have different caregiving experiences due to the availability of health services compared with those outside the program. A related limitation is that participants who were not willing to participate in the survey were also excluded. Second, as the caregivers in this study were PLSs' family members (informal caregivers), our findings cannot be extrapolated to all caregivers of PLSs. Third, the cross-sectional design of this study did not allow for investigating causal relationships among the variables, which should be further examined using longitudinal data. Fourth, a number of family caregivers in our sample had been caring for their loved one with schizophrenia for a long time, which may be different for caregivers of PLSs after a first episode. Fifth, given that the ZBI is a non-specific scale for the evaluation of caregiver burden, this instrument may not fully reflect the caregiver burden in schizophrenia.

## Conclusions

The current study demonstrated that caregiving knowledge and skills were positively related to less stress, anxiety, and depression symptoms. Also, family caregivers with a higher level of caregiving knowledge and skills experienced more caregiving rewarding feelings and were more willing to adopt positive coping strategies in the face of difficulties and challenges. These findings inform future psychoeducational interventions to enhance family caregivers' knowledge and skills of caregiving to improve their psychological well-being and promote active coping.

## Data Availability Statement

The raw data supporting the conclusions of this article will be made available by the authors, without undue reservation.

## Ethics Statement

The studies involving human participants were reviewed and approved by The Institutional review board of Xiangya School of Public Health, Central South University. The patients/participants provided their written informed consent to participate in this study.

## Author Contributions

All authors have made substantial contributions to the study conception and design, data collection and analysis, as well as to the development and editing of the manuscript. ZZ, RL, and YY contributed to the conception and design of the study. ZZ, YW, PF, TL, and YY contributed to the research conduction and data collection. ZZ and YW contributed to data analyses. PF, TL, JT, RL, and YY contributed to data interpretation. ZZ drafted the article while YW, PF, TL, JT, RL, and YY critically appraised it and revised it. All authors approved the final version of manuscript for submission and publication and agreed to be accountable for all aspects of the work.

## Conflict of Interest

The authors declare that the research was conducted in the absence of any commercial or financial relationships that could be construed as a potential conflict of interest.
