# Supplementary material for: Associations of Caregiving Knowledge and Skills With Caregiver Burden, Psychological Well-Being, and Coping Styles Among Primary Family Caregivers of People Living With Schizophrenia in China
Source: Front Psychiatry. 2021 May 26;12:631420. doi: 10.3389/fpsyt.2021.631420 (PMC8187614; doi:10.3389/fpsyt.2021.631420)
Supplement: Supplementary file 1 [file Table_1.DOCX]

| **Supplementary Table 1.** The Knowledge and Skills of Caregiving Scale used in this study and the results of KSCS scores with respect to each item. | | | | | |
| --- | --- | --- | --- | --- | --- |
| Items | Median (IQR) | Number (%) of response per Likert point | | | |
|  |  | 0 | 1 | 2 | 3 |
| Symptoms of the patients with schizophrenia | 2 (2-2) | 17 (4.30) | 62 (15.70) | 224 (56.71) | 92 (23.29) |
| Medicine for the treatment of schizophrenia | 2 (2-3) | 18 (4.56) | 65 (16.46) | 191 (48.35) | 121 (30.63) |
| Side-effects of the medicine | 2 (1-2) | 35 (8.86) | 89 (22.53) | 202 (51.14) | 69 (17.47) |
| How to care for the patients | 2 (2-2) | 7 (1.77) | 55 (13.92) | 238 (60.25) | 95 (24.05) |
| How to control the patients’ symptoms | 2 (1-2) | 14 (3.54) | 85 (21.52) | 222 (56.20) | 74 (18.73) |
| KSCS, Knowledge and Skills of Caregiving Scale; IQR, interquartile range.  Four-point Likert-type KSCS scale: 0, do not understand; 1, almost do not understand; 2, partially understand; 3, totally understand. | | | | | |
